# Gallbladder Cancer or Diffuse Xanthogranulomatous Cholecystitis: A Case of Management Dilemma During Elective Cholecystectomy With Unexpected Severe Mass-Like Pericholecystic Fibrosis and Inflammation

**DOI:** 10.7759/cureus.43375

**Published:** 2023-08-12

**Authors:** Emily R Littman, Charanjeet Singh, Vladimir Neychev

**Affiliations:** 1 Medical School, University of Central Florida College of Medicine, Orlando, USA; 2 Pathology, AdventHealth Winter Park, Winter Park, USA; 3 Surgery, University of Central Florida College of Medicine, Orlando, USA

**Keywords:** pericholecystic inflammation, tumor marker, surgery general, laporoscopic cholecystectomy, xanthogranulomatous cholecystits

## Abstract

A 52-year-old man was scheduled to undergo an elective laparoscopic cholecystectomy for an increasingly symptomatic cholelithiasis. The pre-operative diagnosis was established clinically and confirmed with ultrasonography (US), showing gallstones and thickened gallbladder wall. Intraoperatively, extensive dense adhesions of the omentum to the entire subdiaphragmatic surface of the liver and the diaphragm were encountered. The adhesions of the omentum and colon were completely obscuring the Morrison’s space with cartilage-like consistency at the supposed anatomical projection of the gallbladder fundus. Due to these unexpected pathological findings and uncertain disease biology, a decision was made to abort and re-schedule the surgery after obtained tissue biopsy results, magnetic resonance cholangiopancreatography (MRCP), and tumor markers carbohydrate antigen 19-9 (CA 19-9), carcinoembryonic antigen (CEA), and alpha fetoprotein (AFP) were available. CA 19-9 was found elevated 10-fold, while AFP and CEA levels were within normal limits. A follow-up cholecystectomy was performed, and final pathology revealed diffuse xanthogranulomatous cholecystitis (XC) and extensive inflammatory changes, adhesions, and fibrosis and no malignancy. The patient tolerated the procedure well and was discharged home on day two after surgery. His follow-up examination was unremarkable. Distinguishing between XC and gallbladder carcinoma is important to appropriately guide management and treatment.

## Introduction

Diseases involving the gallbladder account for up to 6% of men and 9% of women with gallstones in the United States [1,2]. Xanthogranulomatous cholecystitis (XC), an uncommon variant of cholecystitis, is characterized by inflammation of the gallbladder with a focal or more-diffuse accumulation of fibrous tissue and lipid-laden macrophages in the gallbladder wall. While focal XG does not usually cause significant pericholecystic inflammation, diffuse XC may lead to direct involvement of surrounding tissues and organs by an extensive, tumor-like inflammatory fibrosis. In rare cases of diffuse XC, the advanced inflammatory changes can be associated with the elevation of tumor markers such as carcinoembryonic antigen (CEA), carbohydrate antigen 19-9 (CA19-9), and alpha fetoprotein (AFP) that makes it virtually indistinguishable from advanced gallbladder carcinoma or cholangiocarcinoma. While benign, the inflammatory changes associated with diffuse XC lead to thickening of involved tissues and mimic malignancy along with many pathophysiologic features and complications such as tumor formation and jaundice [3,4]. Even though diffuse XC is rare, accounting for only 5.2% of resected specimens [5,6], early identification, cholecystectomy and histologic examination are paramount to mitigate misdiagnosis and patient distress. Our case of diffuse XC presented a significant diagnostic and management challenge due to increased tumor marker levels and intraoperative findings highly suggestive of advanced carcinoma.

## Case presentation

A 52-year-old man presented to surgery clinic for evaluation and consideration for possible cholecystectomy following a two-week history of non-radiating, intermittent right-sided abdominal pain. He had an unremarkable surgical history and prior medical history significant for benign prostatic hyperplasia, gastroesophageal reflux disease, Barrett’s esophagus, and asthma. The patient admitted that for a long time he has had more or less constant right abdominal flank pain with dull and sharp aches exacerbated by coughing, deep breaths, or sitting up in bed and leaning forward. The patient denied any associated nausea, vomiting, diarrhea, constipation, fevers, or changes in the characteristics of the pain with respect to food intake. Family history was negative. The patient was previously diagnosed with symptomatic cholelithiasis; however, no intervention was suggested at the time.

At initial evaluation, his vital signs were within normal limits with a body temperature of 98.5°F, a pulse rate of 74 beats per minute, a respiratory frequency of 18 breaths per minute, and a blood pressure of 148/75mmHg. Abdominal examination revealed a soft, non-tender and non-distended abdomen with normal bowel sounds and negative Murphy sign. A 2-cm, non-reducible umbilical hernia was noted. Complete blood count (CBC) and comprehensive metabolic panel (CMP) laboratory results were unremarkable except for elevated total alkaline phosphatase (ALP) (155 U/L). Alanine aminotransferase (ALT) (9 U/L), aspartate aminotransferase (AST) (12 U/L), and total bilirubin (0.7 mg/dL) were within normal limits. A right upper quadrant ultrasound (US) showed cholelithiasis with a thickened gallbladder wall. 

**Figure 1:**
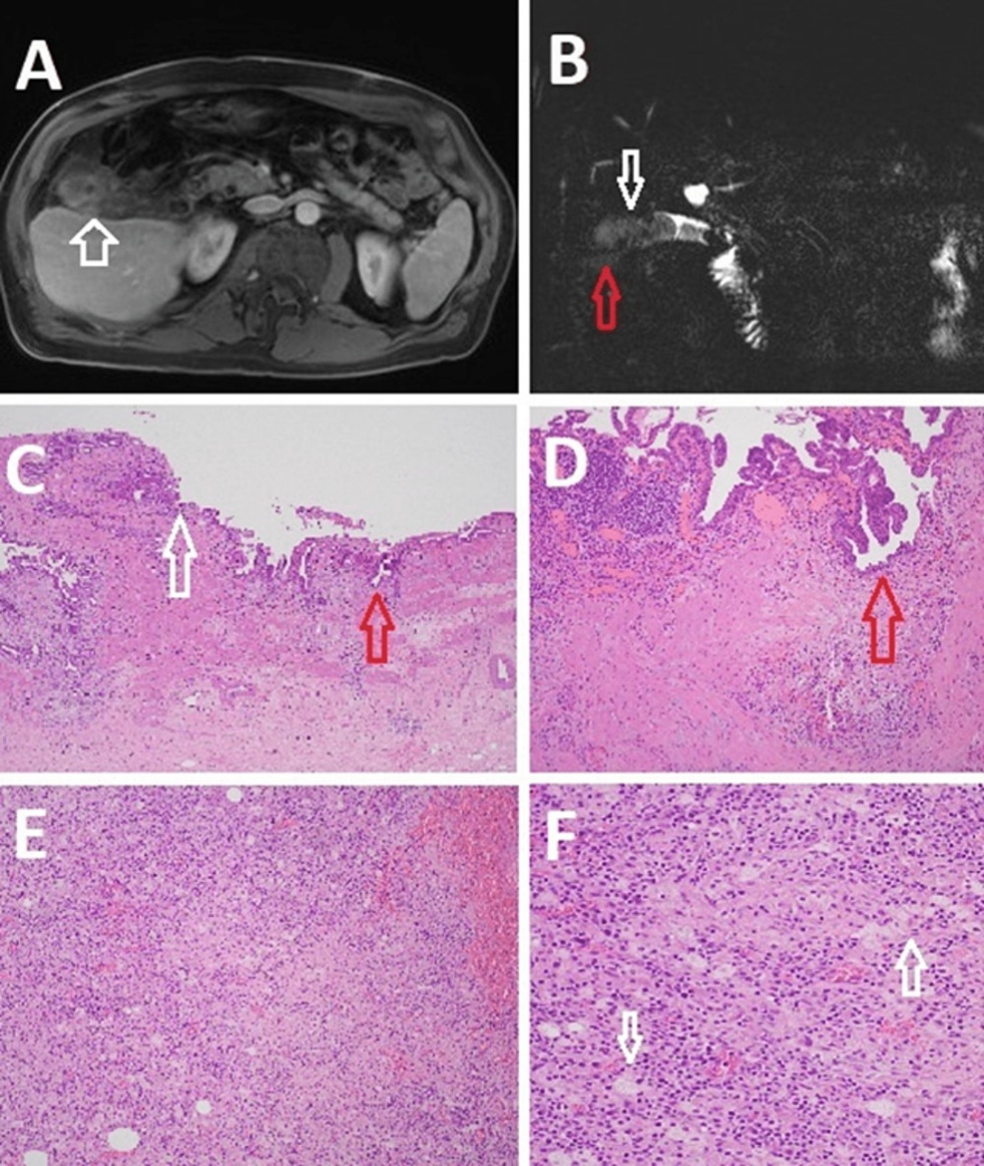
Imaging and surgical pathology of gallbladder specimen A. Axial MRI/MRCP plane; and B. Coronal MRI/MRCP plane, showing an irregular heterogeneous mass occupying the gallbladder (white arrow) with possible contained perforation involving the gallbladder fundus (red arrow); C. Low power image of hematoxylin and eosin stained section of normal-appearing gallbladder mucosa (white arrow) and neighboring areas of ruptured Rokitansky-Aschoff sinuses (red arrow); and D. High power image showing areas of adenomyosis with deep, ruptured Rokitansky-Aschoff sinuses penetrating the muscle layer (red arrow); E. Low power image of hematoxylin and eosin stained section showing foci of crowding of foamy macrophages (xanthoma cells); and F. High power image of foreign body granulomas consisting of cholesterol (white arrows). MRI: magnetic resonance imaging, MRCP: magnetic resonance cholangio pancreatography

Surgical consultation led to recommendation of an elective laparoscopic cholecystectomy. Initial intraoperative inspection of the abdomen revealed extensive adhesions involving the omentum and colon along with the diaphragmatic and inferior surfaces of the liver completely obscuring the Morrison’s space. A meticulous dissection was undertaken to free up the diaphragmatic liver surface from adhesions; however, the adhesions and fibrotic tissue at the projection of the gallbladder fundus were extremely dense with cartilage-like consistency that raised suspicion for possible malignancy. Pathology was consulted intraoperatively and they recommended a tissue biopsy of the fibrotic for histopathology. Due to these unexpected intraoperative findings and since the surgery team was not prepared for possible more extensive resection, a decision was made to defer surgery pending pathology results and to order further workup, including abdominal MRI/MRCP as well as tumor markers CA 19-9, AFP, and CEA. The pathology report returned negative for neoplasm; however, omental tissue demonstrated evidence of chronic lymphoplasmacytic infiltration. CA 19-9 was found elevated to 282 U/mL (normal limit <34 U/mL), AFP and CEA were within normal limits 2.3 ng/mL (normal limit <40 ng/mL) and 0.9 ng/mL (normal limit <5 ng/mL), respectively. The MRI of the abdomen with MRCP revealed cholecystitis with impacted large stone in the gallbladder neck and possibly contained perforation involving the gallbladder fundus. There was gallbladder wall thickening, diffuse pericholecystic soft tissue inflammation with mild biliary ductal dilatation of uncertain etiology without evidence of choledocholithiasis (Figure 1A, 1B). 

Given the workup findings, differential diagnoses included diffuse XC, gallbladder carcinoma (GC), or cholangiocarcinoma. Sixteen days after his first surgery, a decision was made to proceed with a re-do laparoscopic cholecystectomy prepared for exploratory laparotomy and possible more or less extensive surgical resection of the extrahepatic biliary tree and the liver. Intraoperatively, the diaphragmatic liver area was still free of adhesions. Significant inflammatory and fibrotic changes diffusely involving the entire Morison’s space were again encountered. A very careful dissection of the dense fibrotic adhesion of the omentum and colon to the projected fundus of the gallbladder was undertaken that allowed us very slowly to free up the anterior surface of the gallbladder wall. An area of possible perforation in the fundus of the gallbladder draining infected bile was encountered that was evident of gallbladder empyema. The adhesion of the duodenum to the infundibulum of the gallbladder and surrounding structures were less dense and inflamed, which allowed us to identify the gallbladder neck and the proximal cystic duct with the cystic artery. Cholecystectomy was achieved laparoscopically after needle aspiration of purulent material due to infection and associated abscess formation. Surgical pathology revealed marked acute calculous cholecystitis, with transmural abscess and xanthomatous reaction, ulceration, fibrosis, and extensive adhesions (Figure 1C-1F). The dependent lobulated omental fat showed fat necrosis, and focal fibrous reaction. There was no malignancy identified.

Post-operatively, the patient recovered well and was discharged home on day two after surgery without complication. The patient did not report any abdominal pain, issues or any other complaints on his two-week follow-up visit in clinic. His labs including CBC and CMP were within normal limits and the tumor marker CA 19-9 went down to normal postoperatively 12 U/mL (normal limit <34 U/mL). 

## Discussion

While the pathogenesis of diffuse XC is not fully understood, it is suspected that gallstones may cause increased intraluminal pressure, leading to ruptured Rokitansky-Aschoff sinuses, such as in the present case [7]. This may cause bile extravasation into the gallbladder wall which is then engulfed by activated fibroblasts and macrophages [8]. These contribute to the gallbladder wall inflammatory reaction and fibrosis seen in diffuse XC [8]. In cases where inflammation is present and diagnosis is uncertain, the use of tumor markers can prove beneficial. These tests often yield quick results; however, some tumor markers may rise in both benign and malignant conditions [9].

Diffuse XC can present with a wide spectrum of otherwise nonspecific symptoms including fevers, nausea, vomiting, jaundice, right upper quadrant pain postprandial pain, and in some cases presents with weight loss in patients [10-13]. The natural history and biology of diffuse XC make it virtually indistinguishable from that of GC [13,14]. As shown in our case, the fibrosis and inflammation associated with diffuse XC can completely obscure the interface between the liver and gallbladder and involve surrounding soft tissues and organs, that may be virtually indistinguishable from GC [7,14].

These changes often result in similarities on diagnostic imaging (US, MRI, and CT), including diffuse gallbladder wall thickening, nodules of low echogenicity, and the presence of low-density bands [7,14]. There are similarities in the laboratory studies, as well, and both XC and GC can present with elevated tumor markers [15]. Furthermore, the possibility for concomitant XC and GC makes it virtually impossible to have a definitive diagnosis prior to the final pathology results [14].

The mechanisms that cause elevations in tumor marker CA 19-9 are not completely understood. CA 19-9 is known to be synthesized by many cells in the gastrointestinal tract, including pancreatic, biliary, gastric, colonic, and salivary epithelial cells [16]. Some suggest the elevation of CA 19-9 in benign gallbladder disease to be due to cholangiocytic irritation secondary to increases in biliary ductal pressure from factors such as obstructions, inflammation, and bile flow stasis [16]. This irritation, many believe, causes inflammation and CA 19-9 proliferation. Others, however, believe the rise in CA 19-9 to be attributable to decreased hepatic clearance of biliary mucins due to hepatic dysfunctions or end-stage liver disease. CA 19-9 has been shown to have moderate sensitivity (77-78%) and specificity (81-84%) for the diagnosis of biliary tract cancers [17]. Alongside imaging, CA 19-9 has long proven valuable in diagnosing pancreatic and biliary malignancies. However, CA 19-9 has been shown in some cases, including ours, to be elevated in XC which makes the diagnosis of benign vs malignant pathology even more difficult [9,17].

While it is very challenging to distinguish between XC and GC, it is crucial to guide management and treatment. Helpful approaches include CT, MRI, biopsy, and evaluation of tumor markers. Laparoscopic cholecystectomy has shown to be the gold-standard therapy for benign gallbladder disease and is also the standard treatment for XC [11]. However, the extent of the inflammation and adhesions often seen in patients with XC, leads conversion of surgeries from laparoscopy to open in nearly a third of the cases (26%) [18]. The surgical treatment for locally advanced GC includes radical cholecystectomy with an en blanc resection of the external biliary tree and any involved surrounding organs [19]. In our case, we proceeded with laparoscopic cholecystectomy because the additional workup analysis including intraoperative biopsy and tumor markers demonstrated histopathology negative for malignancy and elevations only in CA 19-9 with normal CEA and AFP.

## Conclusions

This case highlights the importance of intraoperative team dynamics and the decision-making process. When surgeons find themselves in doubt for possible but unexpected gallbladder malignancy during an elective cholecystectomy case, it is a valuable option to consider postponing the completion of the surgical procedure until additional workup and careful analysis of the results are performed. Increasing awareness of XC will better ensure this rare condition remains a differential diagnosis and thus expedite the clinical workup and promote patient wellbeing.
